# Laboratory biomarker as an alternative to low-dose computed tomography for the diagnosis of suspected appendicitis? Circulating fibrocyte percentage and neutrophil–lymphocyte ratio are accurate biomarkers of uncomplicated and complicated appendicitis: a prospective cohort study

**DOI:** 10.1097/JS9.0000000000000378

**Published:** 2023-04-03

**Authors:** Rui Zhang, Zhe-Ming Zhao, Chun-Dong Zhang

**Affiliations:** a Department of Colorectal Surgery, Cancer Hospital of China Medical University, Liaoning Cancer Hospital and Institute; b Department of Gastrointestinal Surgery, The Fourth Affiliated Hospital, China Medical University, Shenyang, China

With great interest, we read a recent study by Mohamed *et al.* 1. In this study, Mohamed and colleagues reported that a novel laboratory biomarker, that is, circulating fibrocyte percentage had high diagnostic accuracy in the diagnosis of uncomplicated appendicitis. Besides, they reported that the neutrophil–lymphocyte ratio had similarly high diagnostic accuracy in the diagnosis of complicated appendicitis. This topic is clinically important, providing an important message that laboratory biomarkers may have the ability to diagnose appendicitis accurately without any radiation exposure, potentially reducing lifelong cancer risk, especially for adolescents and young adults. We would like to share some opinions.

Acute appendicitis is one of the most common surgical emergencies. Despite the high incidence of appendicitis, accurate diagnosis of suspected appendicitis has long been challenging, with a high rate of negative (unnecessary) appendectomies in clinical practice. Mohamed and colleagues have tried to identify laboratory biomarkers for diagnosing suspected appendicitis, which is a very important topic in a clinical setting. However, there was no sample size calculation of the minimum number of samples required in this study. The authors applied a limited number of participants in the normal appendix group (*n*=12), complicated appendicitis group (*n*=12), and uncomplicated appendicitis group (*n*=24)^1^. Such a small number may produce biased results; therefore, more participant number is needed.

Besides, they reported relatively high diagnostic accuracy of circulating fibrocyte percentage [area under the curve (AUC), 0.83; sensitivity, 72.7%; specificity, 83.3%] in the diagnosis of uncomplicated appendicitis, and similarly high diagnostic accuracy of neutrophil–lymphocyte ratio in the diagnosis of uncomplicated appendicitis (AUC, 0.84; sensitivity, 75.5%; specificity, 83.3%)^1^. However, we should be cautious that both high diagnostic accuracy rates were based on the best cutoff values identified in the training cohort, and the diagnostic performance of a biomarker should be evaluated by at least one independent validation cohort^2^.

Multiple types of computed tomography (CT), have shown similarly high diagnostic accuracy in the diagnosis of appendicitis, with a pooled high sensitivity of 89–97% and specificity of 93–95%^3^, thus reducing the number of negative appendectomies. As mentioned by Mohamed and his colleagues, radiation exposure was one of the main limitations of CT imaging^1^, and exposures to high dosage of radiation may increase lifelong cancer risk, particularly for adolescents and young adults. However, if we can decrease the radiation dose of CT to an acceptable level, for example, lose-dose CT, then the cancer risk caused by CT radiation exposure will be largely decreased. A single-blind, noninferiority clinical trial reported that low-dose CT (3.5%) was noninferior to standard-dose CT (3.2%) in terms of negative appendectomy rates (difference, 0.3%; 95% CI: −3.8 to 4.6) in young adults with suspected appendicitis^4^. Consistently, Kim *et al.* 5 reported that low-dose CT (AUC, 0.96; sensitivity, 90%; specificity, 92%) had comparable diagnostic performance to standard-dose CT (AUC, 0.97; sensitivity, 89%; specificity, 94%) for the diagnosis of appendicitis in young adults. Furthermore, a pragmatic, multicenter, noninferiority RCT with 3074 adolescents and young adult participants further strengthened the evidence of justifying the use of low-dose CT instead of standard-dose CT in adolescents and young adults with suspected appendicitis^6^, and the radiation dose of appendiceal CT can be reduced to 2 mSv without impairing clinical outcomes^7^.

Overall, there is still no enough evidence that laboratory biomarkers could act as an alternative to CT imaging. Lose-dose CT should still be preferred for the diagnosis of suspected appendicitis in current clinical practice^8^. To prevent unnecessary appendectomies, diagnostic accuracy should still be considered preferentially. However, we shall also pay attention to the following points, including, clinical timeliness, accessibility in regional or rural hospitals, cost-effectiveness, and potential risk.

## Ethical approval

Not applicable.

## Sources of funding

C.-D.Z. was partly supported by the China Scholarship Council (201908050148). R.Z. was partly supported by the National Natural Science Foundation of China (61976249). These funding sources had no role in the study design, in the collection, analysis, and interpretation of data, in the writing, and in the decision to submit the article.

## Author contribution

R.Z.: writing and editing. Z.-M.Z.: editing and revision. C.-D.Z.: conceptualization, writing, editing, revision, and submission.

## Conflicts of interest disclosure

The authors declare that they have no financial conflict of interest with regard to the content of this report.

## Research registration unique identifying number (UIN)

Not applicable.

## Guarantor

Chun-Dong Zhang.

## Data availability statement

The data supporting the findings of this study are available from the corresponding author upon reasonable request.

## Provenance and peer review

Not applicable.

## References

[1] Mohamed Z Peter O’L Miranda K . Circulating fibrocyte percentage and neutrophil–lymphocyte ratio are accurate biomarkers of uncomplicated and complicated appendicitis: a prospective cohort study. Int J Surg 2023;109:343–351. 37093074 10.1097/JS9.0000000000000234 PMC10389644

[2] Zhang CD Takeshima H Sekine S . Prediction of tissue origin of adenocarcinomas in the esophagogastric junction by DNA methylation. Gastric Cancer 2022;25:336–345. 34557982 10.1007/s10120-021-01252-y

[3] Rud B Vejborg TS Rappeport ED . Computed tomography for diagnosis of acute appendicitis in adults. Cochrane Database Syst Rev 2019;2019:CD009977. 31743429 10.1002/14651858.CD009977.pub2 PMC6953397

[4] Kim K Kim YH Kim SY . Low-dose abdominal CT for evaluating suspected appendicitis. N Engl J Med 2012;366:1596–1605. 22533576 10.1056/NEJMoa1110734

[5] Kim SY Lee KH Kim K . Acute appendicitis in young adults: low- versus standard-radiation-dose contrast-enhanced abdominal CT for diagnosis. Radiology 2011;260:437–445. 21633052 10.1148/radiol.11102247

[6] Cho J Lee S Min HD . Final diagnosis and patient disposition following equivocal results on 2-mSv CT vs. conventional-dose CT in adolescents and young adults with suspected appendicitis: a post hoc analysis of large pragmatic randomized trial data. Eur Radiol 2021;31:9176–9187. 33993331 10.1007/s00330-021-08020-7

[7] LOCAT Group. Low-dose CT for the diagnosis of appendicitis in adolescents and young adults (LOCAT): a pragmatic, multicentre, randomised controlled non-inferiority trial. Lancet Gastroenterol Hepatol 2017;2:793–804. 28919126 10.1016/S2468-1253(17)30247-9

[8] Aly NE McAteer D Aly EH . Low vs. standard dose computed tomography in suspected acute appendicitis: Is it time for a change? Int J Surg 2016;31:71–79. 27262882 10.1016/j.ijsu.2016.05.060

